# Overview of distinct N6-Methyladenosine profiles of messenger RNA in osteoarthritis

**DOI:** 10.3389/fgene.2023.1168365

**Published:** 2023-05-09

**Authors:** Yang Yu, Shitao Lu, Yu Li, Jianzhong Xu

**Affiliations:** Department of Orthopedics, The First Affiliated Hospital of Zhengzhou University, Zhengzhou, China

**Keywords:** osteoarthritis, m6A methylation, methylome profile, WGCNA, merip-seq

## Abstract

Although N6-methyladenosine (m6A) modification is closely associated with the pathogenesis of osteoarthritis (OA), the mRNA profile of m6A modification in OA remains unknown. Therefore, our study aimed to identify common m6A features and novel m6A-related therapeutic targets in OA. In the present study, we identified 3962 differentially methylated genes (DMGs) and 2048 differentially expressed genes (DEGs) using methylated RNA immunoprecipitation next-generation sequencing (MeRIP-seq) and RNA-sequencing. A co-expression analysis of DMGs and DEGs showed that the expression of 805 genes was significantly affected by m6A methylation. Specifically, we obtained 28 hypermethylated and upregulated genes, 657 hypermethylated and downregulated genes, 102 hypomethylated and upregulated genes, and 18 hypomethylated and downregulated genes. The differential gene expression analysis based on GSE114007 revealed 2770 DEGs. The Weighted Gene Co-expression Network Analysis (WGCNA) based on GSE114007 identified 134 OA-related genes. By taking the intersection of these results, ten novel aberrantly expressed, m6A-modified and OA-related key genes were identified, including SKP2, SULF1, TNC, ZFP36, CEBPB, BHLHE41, SOX9, VEGFA, MKNK2 and TUBB4B. The present study may provide valuable insight into identifying m6A-related pharmacological targets in OA.

## 1 Introduction

Osteoarthritis (OA) is the most common type of arthritis and one of the leading causes of pain and disability worldwide. The primary features of OA include articular cartilage degeneration, synovial inflammation, and subchondral bone sclerosis (Peat and Thomas, 2021). Despite advances in OA treatment, there are still challenges in attenuating its devastating effects. One of these challenges is the lack of methods to reverse cartilage degradation and inhibit progression in advanced OA. The current effective treatment, joint replacement, causes financial, physical and psychological burden. To develop new therapies, it is urgently needed to elucidate the underlying molecular mechanism of OA pathogenesis (Quicke et al., 2022).

N6-methyladenosine (m6A) modification is the most common RNA epigenetic modification in mammalian cells. The epigenetic regulation of m6A methylation regulates gene expression and translation, therebyaffecting cell development and differentiation. Accumulating evidence indicates that m6A modification participates in the regulation of critical biological processes and pathogenesis of numerous human diseases (Zhao et al., 2020; Jiang et al., 2021). A variety of m6A-modified mRNAs have the potential to serve as novel biomarkers for diagnosis or therapeutic targets for chemotherapy (Ianniello et al., 2019; Liu K. et al., 2022; Zhang H. et al., 2022; Qiu et al., 2022). Furthermore, the importance of m6A modification is underscored by its evolutionary conservation across species and its presence in all eukaryotic organisms. The modification is also dynamically regulated by a complex interplay of enzymes, readers, and erasers, which further emphasizes its critical role in cellular processes.

Recently, the regulatory role of m6A methylation in OA has garnered increasing attention. The overall m6A modification was found to be elevated in IL-1β treated chondrocytes, and the dysregulation of m6A modification in chondrocytes led to the aberrant expression of genes involved in extracellular matrix (ECM) synthesis and degradation, which plays a critical role in the development and progression of OA (Liu et al., 2019). Moreover, several enzymes involved in m6A modification have been found to be differentially expressed in OA chondrocytes, suggesting that their dysregulation may contribute to the pathogenesis of OA. For example, m6A writer protein METTL3 was found to be upregulated in chondrocytes and associated with ECM degradation and proliferation repression (Ren et al., 2022). The eraser protein FTO was found to be downregulated in chondrocytes, resulting in increased apoptosis and ECM degradation (Yang et al., 2021). Several studies have revealed the pleiotropic roles of m6A modification in OA. M6A modification can regulate OA by mediating cellular senescence (Chen et al., 2022), apoptosis (Liu et al., 2019), inflammation response (Shi et al., 2022), and ECM degradation (Sang et al., 2021). Given the significant impact of m6A modification on chondrocyte metabolism, it is essential to search for and identify more core substrate genes of m6A modification in OA. These m6A-modified genes could be promising therapeutic targets and research focus to further elucidate the underlying mechanism of OA pathogenesis. However, until now, the transcriptome-wide m6A profile, the functions and pathways in which m6A-methylated genes are enriched, and the potential impacts of m6A modifications on mRNA expression in OA remain unknown.

In the present study, we collected three OA knee cartilage tissues and three normal knee cartilage tissues to obtain the first transcriptome-wide m6A modification profile of mRNAs in OA using methylated RNA immunoprecipitation next-generation sequencing (MeRIP-seq). Then, we performed a co-expression analysis to determine the correlation between m6A modification and mRNA expression in OA. Moreover, combining the results of weighted gene co-expression network analysis (WGCNA) based on GSE114007, we screened ten novel m6A-modified key genes in OA.

## 2 Materials and methods

### 2.1 Sample collection

Knee cartilage samples of medial condyle were collected from three patients who underwent knee arthroplasty and three patients who underwent thigh amputation due to trauma. Rheumatoid arthritis and other metabolic diseases were excluded in all patients. Clinical characteristics of included patients were shown in Table 1. Fresh samples were immediately frozen in liquid nitrogen and stored at −80°C to be detected. This study was approved by the institutional ethics board of the First affiliated Hospital of Zhengzhou University (2022-KY-0854-002).

**TABLE 1:** Clinical characteristics of included patients.

Sample	Age	Gender	BMI	Kellgren-Lawrence
				Grade (n)
OA 1	74	female	27.5	4
OA 2	69	female	30.2	4
OA 3	72	female	28.6	4
Normal 1	61	female	23.2	0
Normal 2	53	female	22.4	0
Normal 3	68	female	24.6	1

### 2.2 MeRIP-sequencing

Total RNA was extracted using TRIzol™ Reagent (Invitrogen, 15596018). The concentration of total RNA was measured by Qubit RNA HS assay kit (Invitrogen, Q32852). 100μg total RNA was fragmented into 100-200 nt RNA fragments using 10X RNA Fragmentation Buffer (100 mM Tris-HCl, 100 mM ZnCl2 in nuclease-free H2O). The reaction was stopped by adding 10X EDTA (0.5M EDTA). Methylated RNA immunoprecipitation was performed using EpiTM m6A immunoprecipitation kit (Epibiotek, R1804). Briefly, the fragmented RNA was incubated with anti-m6A monoclonal antibody (Abcam, ab208577) for 3 h at 4°C and then with protein A/G magnetic beads (Invitrogen, 8880210002D/10004D) at 4°C for an additional 2 h to obtain immunoprecipitated RNA fragments. The m6A-enriched RNA was purified using TRIzol™ Reagent (Invitrogen, 15596018). The library was prepared by smart-seq method. Both the input samples without IP and the m6A IP samples were subjected to 150-bp, paired-end sequencing on an Illumina NovaSeq 6000 sequencer.

### 2.3 Data processing

Cutadapt (v2.5) was used to trim adapters and filter for sequences, and the remaining reads were then aligned to the human Ensemble genome GRCh38 (mouse Ensemble genome GRCm38) using Hisat2 aligner (v2.1.0). The m6A peaks on the mRNA of the three OA samples were combined to obtain the m6A peaks of the OA group, and the normal group was treated in the same way. The m6A peaks and the differential m6A peaks were identified using the exomePeak R package (v2.13.2) under the parameters “PEAK_CUTOFF_*p*-value = 0.05, PEAK_CUTOFF_FDR = NA, and FRAGMENT_LENGTH = 200.” The Gene Ontology (GO) project contains three parts: biological processes (BP), molecular functions (MF), and cellular components (CC). Differentially m6A-modified genes were used to perform GO functional analysis to annotate and speculate on the function of these differentially methylated genes. Pathway analysis using the Kyoto Encyclopedia of Genes and Genomes (KEGG) was conducted with differentially methylated genes for annotation and inference of the pathways they could be involved in. GO and KEGG analyses were performed using the clusterprofile R package (v3.6.0) with a *p*-value <0.05 were considered statistically significant. The m6A-RNA-related genomic features were visualized using the Guitar R package (v1.16.0). We identified m6A peaks with a *p*-value <0.05 for the *de novo* motif analysis using homer (v4.10.4). The differential gene expression analysis was performed using the DESeq2 R package with *p*-value <0.05 and fold change >2 as the cutoff criteria in the data processing of RNA-seq of input.

### 2.4 GEO datasets download and gene differential expression analysis

Datasets were downloaded from the Gene Expression Omnibus (GEO, https://www.ncbi.nlm.nih.gov/geo) database. The GSE114007 dataset includes 19 OA knee cartilage samples and 19 normal knee cartilage samples (Fisch et al., 2018). The differential expression analysis was performed using the wilcox. test by the limma package (v3.40.6). Differences were defined as statistically significant when the *p*-value <0.05 and fold change >2.

### 2.5 WGCNA analysis

The gene co-expression networks were performed by the WGCNA package based on the mRNA expression data of GSE114007. Briefly, a weighted correlation network was constructed by establishing a matrix of pairwise correlations between all the mRNAs across the measured samples. Then, the adjacency matrix was constructed using the power β = 13 as the soft-threshold. Next, the connectivity measure per gene was calculated based on the connection strengths with other mRNAs in the network. Subsequently, genes with strong co-expression relationships were acquired as modules by average linkage hierarchical clustering. After that, the gene expression profiles of each module were summarized by the module eigengene (ME), which was the first principal component of each module’s gene expression profile. The module with the highest correlation score was defined as the key module. Hub genes of the key module were identified by calculating the gene significance (GS) and module membership (MM). |GS| > 0.6 and |MM| > 0.8 were set as cutoff criteria. The detailed procedure of the WGCNA is shown in the WGCNA R package (Langfelder and Horvath, 2008).

## 3 Results

### 3.1 Flow chart

**FIGURE 1:**
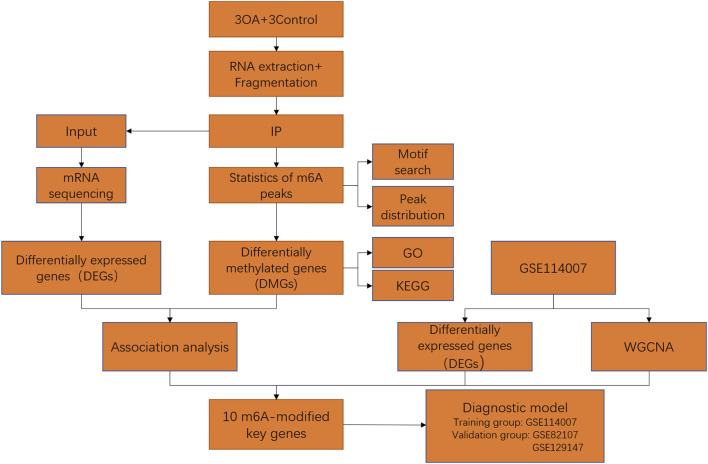
Flow chart of identification of m6A-related key genes and construction of a diagnostic model.

A flow chart of our study is shown in Figure 1.

### 3.2 Common features of the m6A modification of mRNA in OA and normal knee cartilage

**FIGURE 2:**
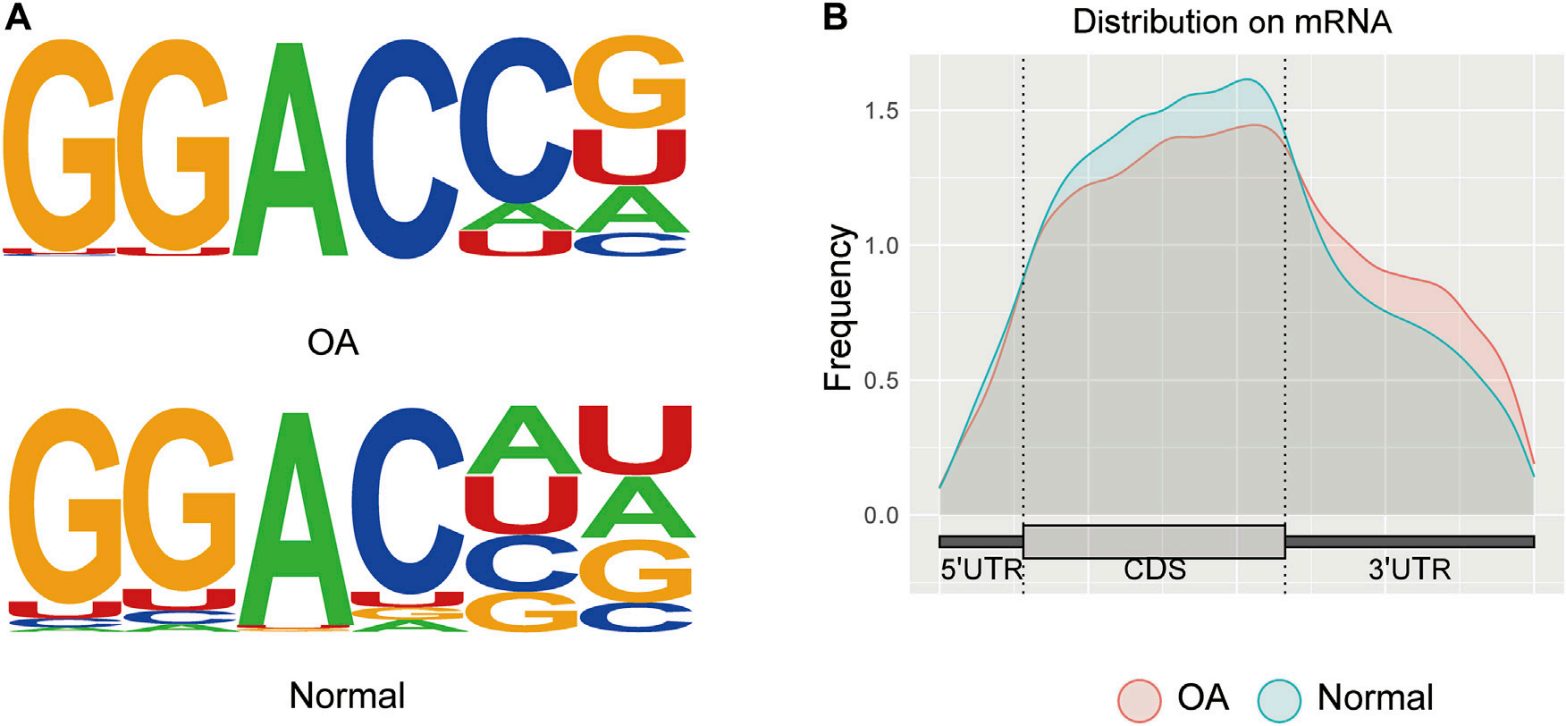
Transcriptome-wide analysis of m6A peaks. **(A)** Common motif enriched in the m6A peak in the mRNA identified in OA and normal cartilage. **(B)** The distribution of m6A peaks in the length of mRNA between OA and normal cartilage.

In this study, six samples were detected for mRNA m6A methylation, including three OA knee cartilage samples and three normal knee cartilage samples. After removing the low-quality data, approximately 42111259 reads were acquired from every OA IP sample, and approximately 43334627 reads were acquired from every normal IP sample. In addition, the consensus sequence GGAC, the common m6A motif described in human diseases, was found to be the most significant peak using MOMER software (Figure 2A). Moreover, the m6A peaks were significantly related to different gene locations in both the OA and control samples: mostly in the 3′ UTR and the CDS region (Figure 2B).

### 3.3 Different m6A modifications between the OA sample and the control sample

**FIGURE 3:**
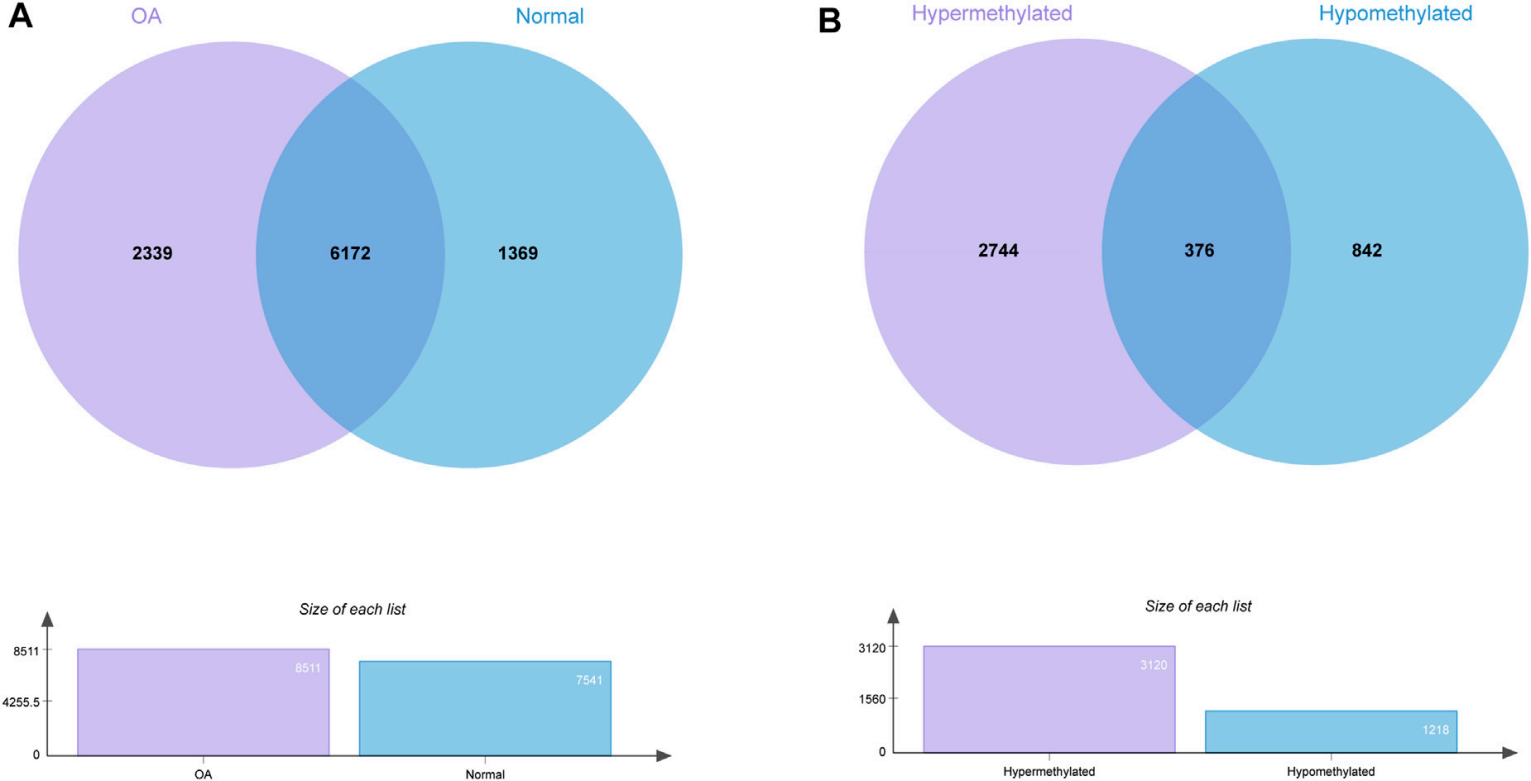
Statistics for genes with m6AI modifications in OA and normal cartilage. **(A)** Number of genes with m6A sites in OA and normal samples. **(B)** Number of genes with hyper- and hypomethylated m6A sites in OA.

**FIGURE 4:**
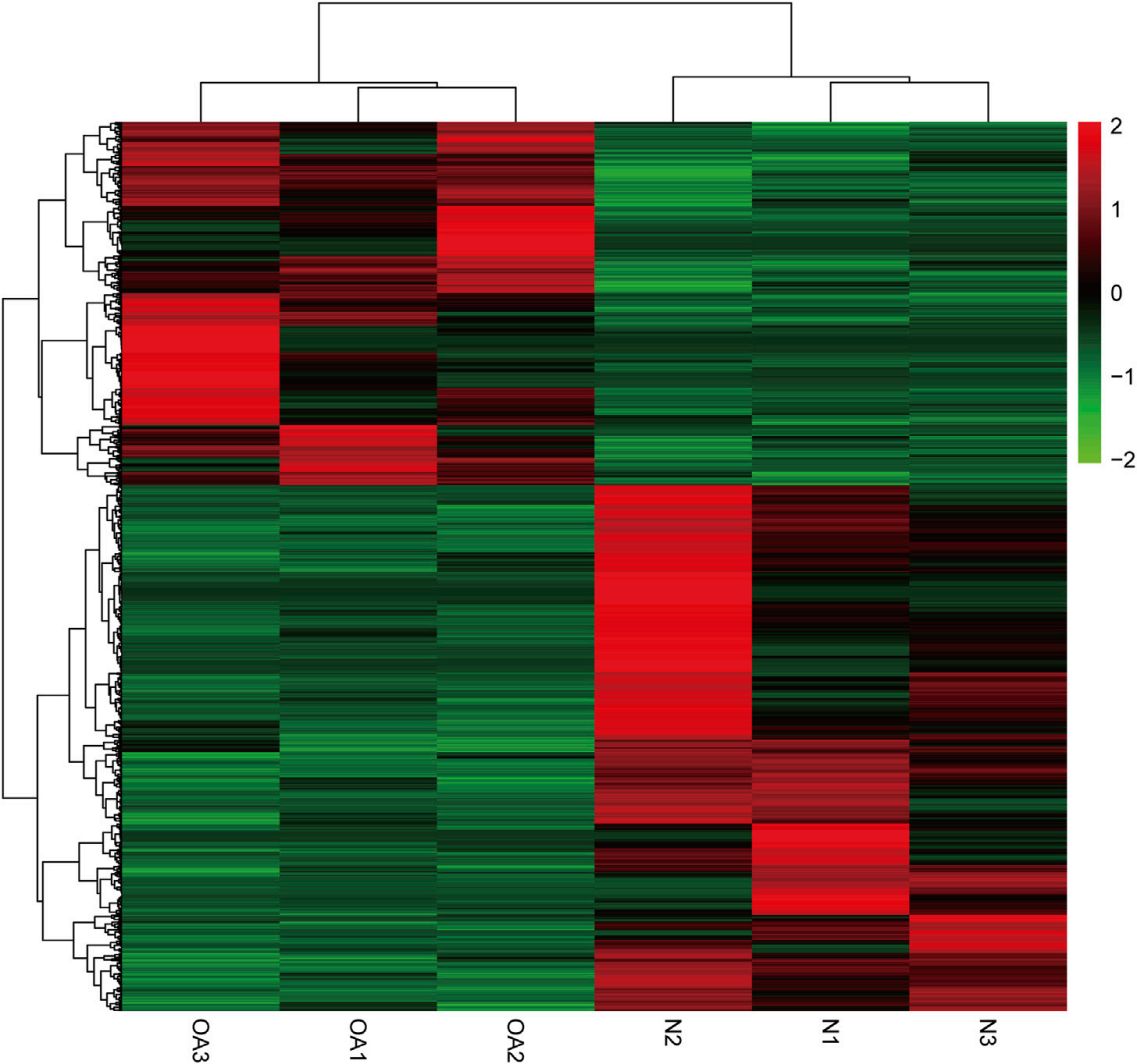
Cluster analysis of m6A methylation in OA and normal knee cartilage. Heatmap of differentially methylated m6A peaks in 3962 DMGs.

We identified 8511 genes with 25295 peaks in the OA sample and 7541 genes with 17474 peaks in the control sample, indicating an overall hypermethylated level in the OA cartilage. On average, 2.97 and 2.31 m6A sites occurred per gene mutation in the OA and normal cartilage, respectively. In addition, 6172 genes were m6A methylated in both OA and control groups (Figure 3A). By comparing the differential m6A modifications in the OA and control samples, we identified 3120 differentially methylated genes (DMGs) containing hypermethylated sites and 1218 DMGs containing hypomethylated sites in the OA sample. Among them, 376 DMGs contained both hypermethylated and hypomethylated sites (Figure 3B, Supplementary Table S1). The results of the methylation heatmap and cluster analyses showed that: there were relative consistencies within the groups and marked differences between the groups (Figure 4). The top ten up- and down-methylated peaks in the OA cartilage and their related information are shown in Table 2.

**TABLE 2:** The Top ten up- and down-methylated peaks in OA cartilage.

Catalog	Gene name	Chrom	Strand	ThickStart	ThickEnd	Log_2_ fc
Up	SNX6	14	-	34605644	34609694	10.4
	NBN	8	-	89958739	89970509	9.98
	RDH10	8	+	73323547	73323787	9.69
	POGLUT1	3	+	119493030	119493211	9.68
	BCL9L	11	-	118897714	118898135	9.64
	MARS	12	+	57516402	57516642	9.48
	NAA15	4	+	139389038	139389219	9.34
	PLEKHH3	17	-	42668122	42668481	9.05
	CAST	5	+	96772824	96773154	8.78
	NUP188	9	+	128995318	128998164	8.75
Down	HMGN5	X	-	81114684	81118434	−9.47
	TCEAL8	X	-	103253649	103253948	−9.19
	RSPO2	8	-	107901104	107958232	−9.12
	SETBP1	18	+	44701517	44701818	−8.89
	SERTAD2	2	-	64636011	64636281	−8.75
	ZC3H13	13	-	45979837	45985340	−8.45
	ISM1	20	+	13299309	13299519	−8.32
	MAP1A	15	+	43522448	43522779	−8.24
	KTN1	14	+	55675839	55678543	−8.16
	FBXL7	5	+	15937967	15938177	−8.15

### 3.4 Gene enrichment analysis of DMGs

**FIGURE 5:**
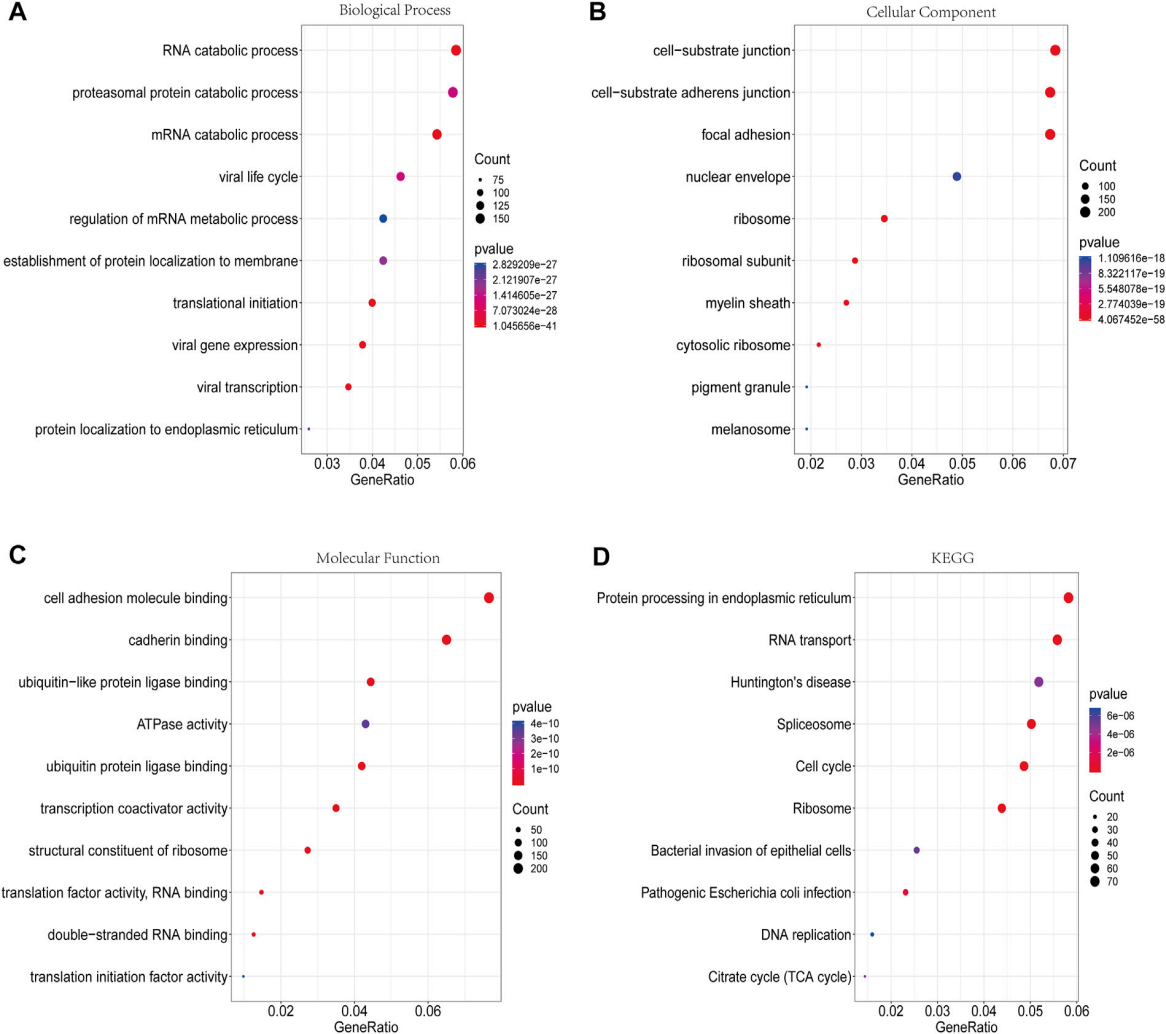
GO functional enrichment analysis and KEGG pathway analysis of DMGs. **(A)** Biological process annotation diagram (Top10). **(B)** Cellular component annotation diagram (Top10). **(C)** Molecular function annotation diagram (Top10). **(D)** KEGG annotation.

The GO analysis revealed that the DMGs were mainly involved in biological processes such as translational initiation, RNA catabolic processes, viral gene expression, and mRNA catabolic processes (Figure 5A). In terms of cellular component, these genes were mainly enriched in cell-substrate junctions, focal adhesions, cell-substrate adherens junctions, and ribosomes (Figure 5B). In terms of molecular function, these genes were mainly engaged in cadherin binding, cell adhesion molecule binding, ubiquitin-like protein ligase binding, and ubiquitin protein ligase binding (Figure 5C). In addition, the KEGG analysis showed that the DMGs were chiefly enriched in pathways including ribosomes, spliceosomes, RNA transport and the cell cycle (Figure 5D).

### 3.5 Analysis of the effects of differential m6A modification of genes on related mRNA expression

**FIGURE 6:**
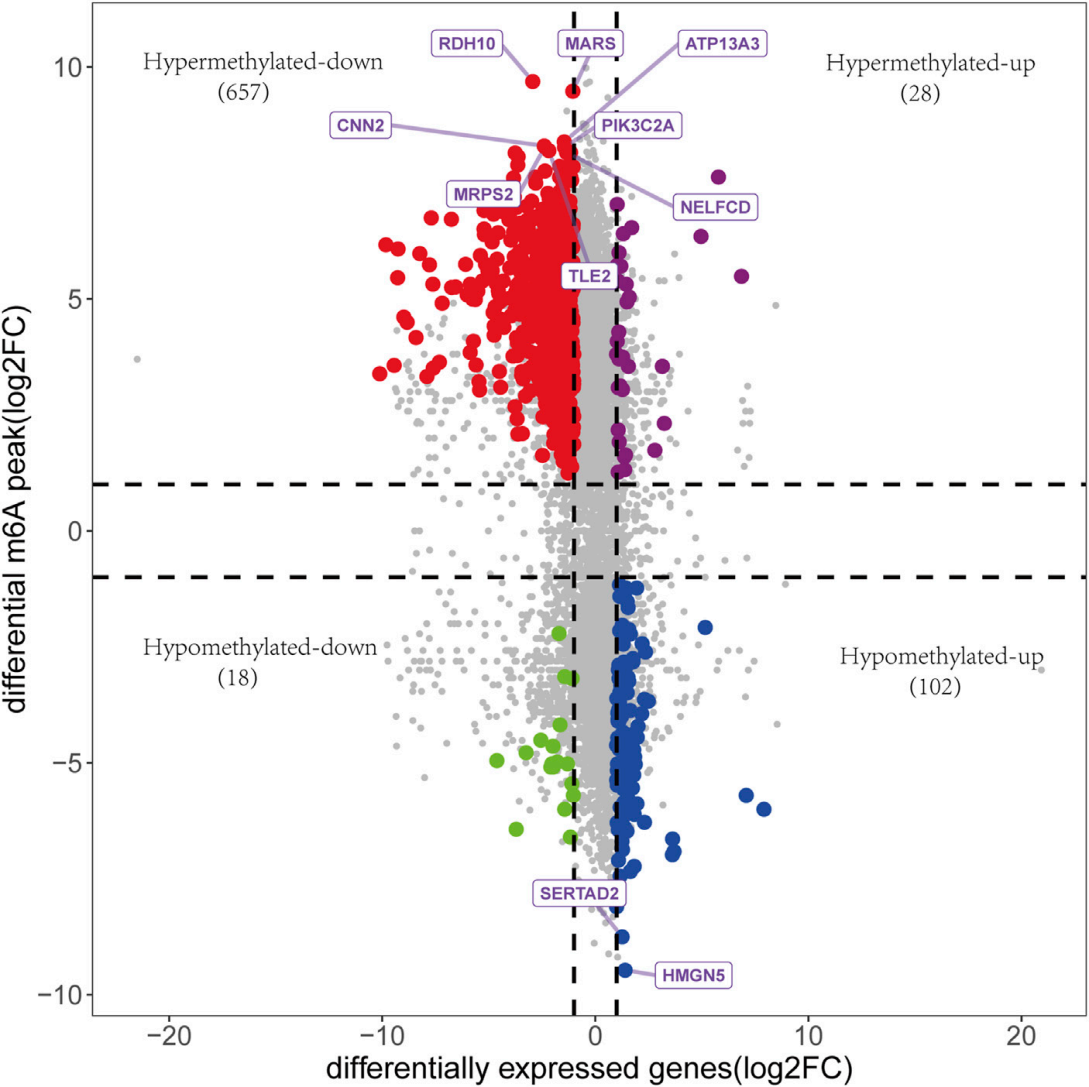
The association analysis between DEGs and DMGs. 805 genes were identified, including 28 hypermethylated and upregulated genes, 657 hypermethylated and downregulated genes, 102 hypomethylated and upregulated genes, and 18 hypomethylated and downregulated genes. Genes with the top 10 m6A modification sites were annotated.

A total of 759 upregulated genes and 1289 downregulated genes were identified from the RNA-seq of input (Supplementary Table S2). The association analysis of these 2048 differentially expressed genes (DEGs) with 3962 DMGs showed that the expression of 805 genes was affected by m6A methylation, including 28 hypermethylated and upregulated genes, 657 hypermethylated and downregulated genes, 102 hypomethylated and upregulated genes, and 18 hypomethylated and downregulated genes (Figure 6, Supplementary Table S3).

### 3.6 Identification of the m6A-modified key genes in OA

**FIGURE 7:**
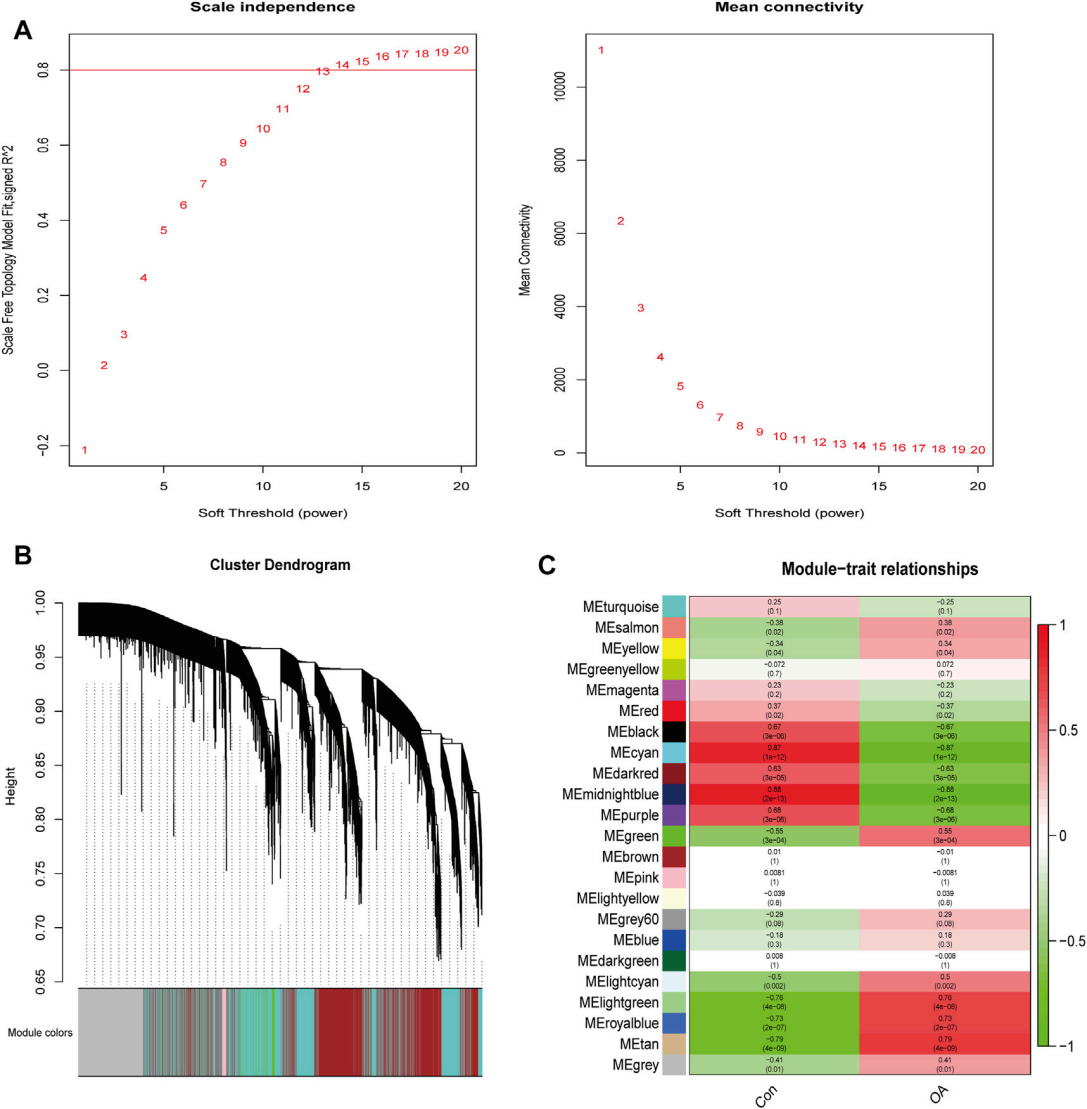
WGCNA analysis. **(A)** Identification of optimal soft threshold power for the co-expression network. **(B)** Clustering dendrogram of genes. **(C)** Module-trait relationships.

**FIGURE 8:**
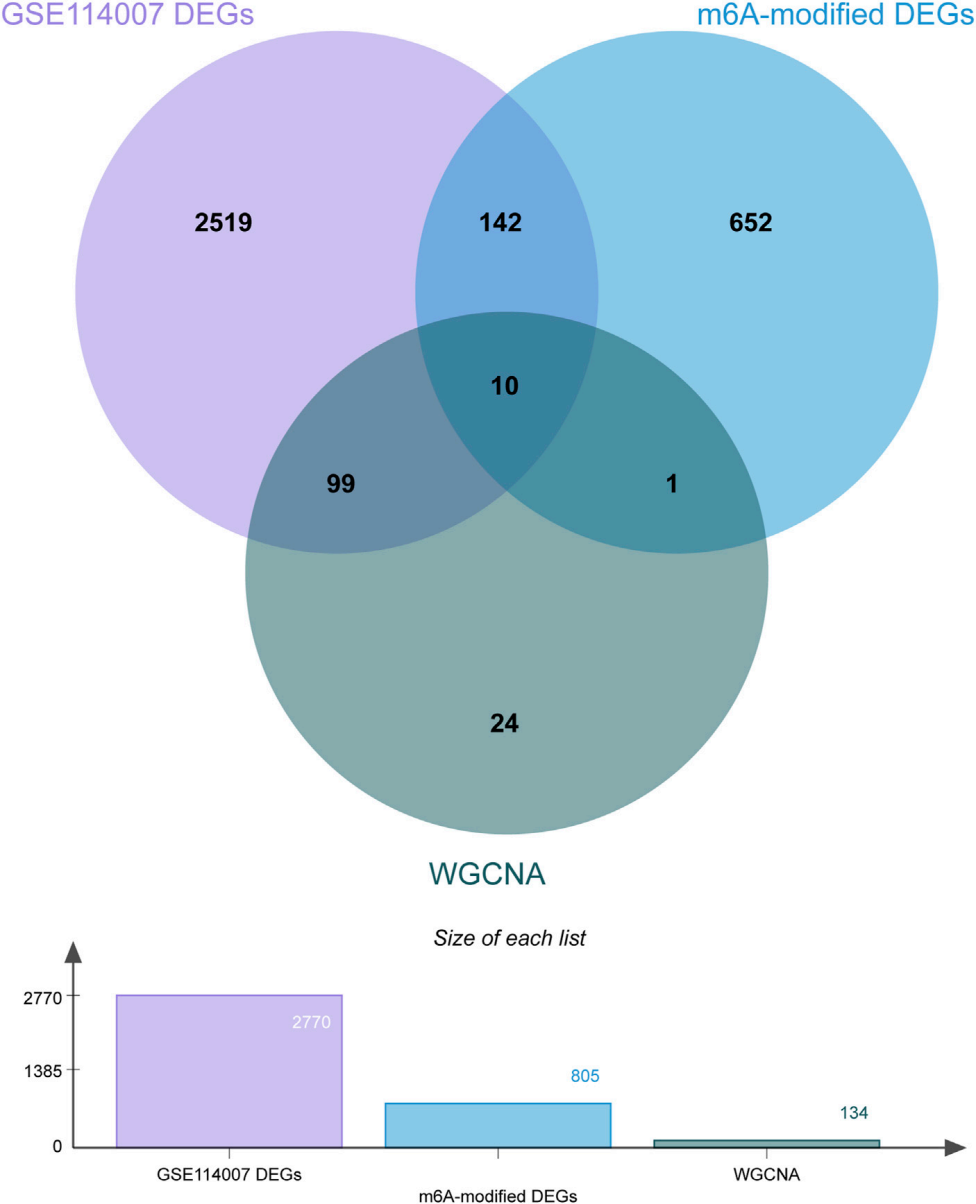
Venn diagram of the WGCNA, DEGs and DMGs. Ten m6A-modified key genes were identified by crossover.

A total of 2770 DEGs, including 1049 downregulated and 1721 upregulated genes, were identified from GSE114007 under the thresholds of |log_2_FC| > 1 and *p* < 0.05 (Supplementary Table S4). In addition, to identify the hub genes correlated with OA, we conducted the WGCNA for the genes gained from GSE114007. When the soft thresholding was set at 13, the scale-free topology fit index reached 0.80 (Figure 7A) and 23 co-expressed gene modules were detected (Figure 7B). The module of midnight-blue showed the strongest significant correlations with OA (*r* = 0.88, *p* = 2e-13) (Figure 7C). A total of 134 genes were screened as hub genes in this module according to |MM|>0.8 and |GS|>0.6 (Supplementary Table S5). Finally, by overlapping the WGCNA results, the DEGs and the DMGs, we identified ten m6A-modified and OA-related key genes, including SKP2, SULF1, TNC, ZFP36, CEBPB, BHLHE41, SOX9, VEGFA, MKNK2 and TUBB4B (Figure 8; Table 3).

**TABLE 3:** Ten m6A-modified and OA-related key genes in OA.

Category	Hypermethylated	Hypomethylated
Upregulated	SKP2	SULF1
		TNC
		ZFP36
		BHLHE41
		SOX9
		VEGFA
Downregulated		
	CEBPB	
	MKNK2	
	TUBB4B	

## 4 Discussion

Despite m6A modification being a hot emerging field in OA pathogenesis with rapidly expanding interest, the full understanding of the molecular mechanisms of m6A modification in OA is still in its infancy. Most relevant studies were designed from the perspective of the m6A regulatory proteins. Among the studied regulators, the “writer” METTL3 is the most well-documented. It was shown that METTL3-mediated m6A modification attenuated the RNA stability of ATG7 and negatively regulated autophagy in the OA fibroblast-like synoviocytes (Chen et al., 2022). Another study showed that METTL3 accelerated OA by regulating the NF-kB signaling pathway and the extracellular matrix (ECM) degradation (Liu et al., 2019). METTL3 also catalyzes the m6A methylation of non-coding RNAs in OA pathogenesis. The expression of LINC00680 could be increased by METTL3 in an m6A-dependent manner in chondrocytes, facilitating OA progression (Ren et al., 2022). A study on temporomandibular joint arthritis showed that METTL3 inhibited apoptosis and autophagy of chondrocytes in inflammation through the m6A/YTHDF1/Bcl2 signaling axis, providing a promising therapeutic strategy for temporomandibular joint arthritis (He et al., 2022).

In our present study, we overviewed the transcriptome-wide m6A profile and obtained abundant m6A-modified mRNAs. These findings might offer researchers valuable options for further research. Additionally, we identified ten m6A-modified key genes through a series of bioinformatics methods, six of which are OA-related, including SULF1, CEBPB, VEGFA, SKP2, SOX9 and TNC. However, none of them has been studied from the m6A perspective in previous literature. Specifically, SULF1 is an extracellular heparan sulfate proteoglycan-specific 6-O-endosulfatasethat can increase type II collagen expression and decrease MMP-13 expression in chondrocytes (Otsuki et al., 2017). In SULF1 knockout mice, MMP-13 were elevated whereas type II collagen and aggrecan were reduced, resulting in more severe spontaneous cartilage degeneration than in wild-type mice (Otsuki et al., 2010). CEBPB is a transcription factor of the basic-leucine zipper class and plays critical roles in proliferation and differentiation of a variety of tissues (Niehrs and Calkhoven, 2020). It enhanced the promoter activity of MMP13, and knockout of CEBPB in mice caused resistance to OA with decreased MMP-13 expression (Hirata et al., 2012). Additionally, CEBPB was identified as a substrate of m6A modification in autoimmune conditions (Bechara et al., 2021) and cholangiocarcinoma (Zhu et al., 2022). VEGFA is an essential member of vascular endothelial growth factor family and a crucial regulator of vasculogenesis and angiogenesis, with multiple signaling pathways and factors involved in the regulation of its expression. M6A modification facilitated the stability of VEGFA by preventing its mRNA degradation in colorectal cancer (Zhang G. et al., 2022; Liu X. et al., 2022). Moreover, in bone mesenchymal stem cells, m6A modification participated in regulating the alternative splicing of VEGFA (Tian et al., 2019). However, how VEGFA is regulated by m6A in OA pathogenesis remains unknown, warranting further research. SKP2, also known as p45, is a kinase-associated protein that participate in cell apoptosis, proliferation, migration, and invasion in multiple types of human cancer (Wang et al., 2012; Cai et al., 2020). In OA pathogenesis, SKP2 was shown to modulate the MAPK signaling pathway and ECM degradation (Feng et al., 2020), and suppressing SKP2 by miR-337-3p could inhibit ubiquitination of DUSP1 and promote chondrocyte proliferation (Jian et al., 2021). SKP2 is an important ligase of ubiquitination, but its relationship with m6A methylation remains unclear. SOX9 is a critical transcription factor and master regulator in chondrogenesis (Song and Park, 2020), promoting the expression of ECM protein during chondrogenic differentiation (Yang et al., 2022). Elevated expression of SOX9 could alleviate the severity of OA, making it a promising therapeutic target in OA (Tong et al., 2021). Nevertheless, the molecular mechanisms regulating SOX9 expression are still largely unknown. In a testicular cancer cell line, phosphorylation of SOX9 promoted its nuclear translocation (Malki et al., 2005). In degenerative human endplate cartilage, METTL3 modulated SOX9 m6A modification and reduced its mRNA stability, thereby inhibiting the expression of the type II collagen (Xiao et al., 2022). These results suggest that the expression of SOX9 is epigenetically regulated. However, the epigenetic regulation of SOX9, including m6A modification, has not been explored in OA pathogenesis. TNC is a large molecular glycoprotein and component of the extracellular matrix with wide involvement in cartilage development and chondrogenesis (Chiquet and Fambrough, 1984). Its expression decreased along with the maturation of chondrocytes and almost disappeared in normal adult articular cartilage (Chevalier et al., 1994). TNC has been reported to induce inflammatory mediators and accelerate cartilage degradation (Patel et al., 2011; Chockalingam et al., 2013), but it also has the potential to repair cartilage defects (Matsui et al., 2018), with elevated expression in OA cartilage indicating a self-repair mechanism (Hattori et al., 2021). The dual roles of TNC may be derived from the versatile nature of this glycoprotein. Although TNC has been widely studied in OA, the regulatory role of m6A modification in TNC has never been addressed. Thus, further studies in the m6A perspective may help to elucidate the underlying mechanism of the two opposing roles of TNC in OA. In addition, four m6A-modified key genes found in our study, including MKNK2, TUBB4B, BHLHE41, and ZFP36, have never been studied in OA pathogenesis and may be valuable for further exploration as potential therapeutic targets according to our results.

Intriguingly, our study revealed that most hypermethylated mRNAs were downregulated, while most hypomethylated mRNAs were upregulated in OA cartilage. This finding may have implications for further understanding the role of m6A modification in OA. M6A modification exerts different functional mechanisms, including mRNA splicing, mRNA translation, mRNA degradation, mRNA stability, and mRNA maturation in different pathological processes (Jiang et al., 2021). Our results indicated that decreased mRNA stability and elevated mRNA decay, which generally result in reduced mRNA expression, might be essential processes involved in the regulatory metabolisms of many m6A-modified mRNAs in OA. On the other hand, m6A-binding proteins can recognize m6A structures and regulate the subsequent biological processes of mRNA. These proteins have been extensively studied in multiple disorders, including the YTH domain family (YTHDF1/2/3 and YTHDC1/2), insulin-like growth factor 2 mRNA-binding proteins (IGF2BP1-3), and heterogeneous nuclear ribonucleoproteins (HNRNPs, hnRNPC, hnRNPG, and hnRNPA2B1) (Feng et al., 2022). Among them, YTHDF2 has been the most well-recognized m6A-binding protein that destabilizes mRNAs and decreases the expression of target genes (Paris et al., 2019; Wang and Lu, 2021). However, the role of YTHDF2 in the pathogenesisof OA is still poorly understood. It could be assumed that YTHDF2 might be an important m6A-binding protein in OA, and further study of YTHDF2 in OA might be promising.

The balance between catabolic and anabolic signaling pathways is critical for maintaining cartilage homeostasis, and its disturbance contributes to OA. By GO and KEGG enrichment analyses, we found that the DMGs in OA cartilage were involved in RNA catabolic processes. This finding suggested that m6A modification affected metabolic processes that reflected the specific activities of the pathogenesis of OA. GO analysis also showed that the DMGs were correlated with the molecular functions of ubiquitin ligase. This is in accordance with previous observations in cancer. In osteosarcoma, an m6A modification disorder led to increased expression of the ubiquitin ligase RNF40, resulting in unchecked replication (Yadav et al., 2022). Ubiquitin ligases have been demonstrated to have an important impact on skeletal homeostasis by regulating the stability and function of various signaling factors (Liang et al., 2020). For instance, the overexpression of PARKIN, an E3 ubiquitin ligase, could accelerate the bone healing process (Zhang et al., 2020). PARKIN could also inhibit inflammation and aging-related metabolic changes in chondrocytes and attenuate OA progression through ubiquitination (Xu et al., 2020). Taken together, m6A RNA methylation may work in concert with other epigenetic mechanisms, such as ubiquitination, to participate in the pathogenesis of OA.

It should be acknowledged that our study has several limitations. First, the small sample size may affect the accuracy of the results. Second, our results are mainly based on high-throughput sequencing and bioinformatics analysis, and further molecular experiments are needed to verify our hypotheses.

## 5 Conclusion

In this study, we outlined the patterns and characteristics of m6A modification in OA and normal cartilage tissues for the first time. We obtained abundant m6A-modified sites in mRNAs and observed significant differences between the two groups. Then we identified ten m6A-modified key genes in OA pathogenesis by in-depth bioinformatics analysis. We believe our study offers a valuable reference for further investigation of m6A-related therapeutic targets in OA.

## Data Availability

The datasets presented in this study can be found in online repositories. The names of the repository/repositories and accession number(s) can be found in the article/Supplementary Material.
